# Properties of Table Tennis Blade from Sorghum Bagasse Particleboard Bonded with Maleic Acid Adhesive at Different Pressing Temperatures and Times

**DOI:** 10.3390/polym15010166

**Published:** 2022-12-29

**Authors:** Jajang Sutiawan, Rudi Hartono, Dede Hermawan, Yusuf Sudo Hadi, Deded Sarip Nawawi, Imam Busyra Abdillah, Alifah Syahfitri, Sukma Surya Kusumah, Danang Sudarwoko Adi, Wida Banar Kusumaningrum, Muhammad Adly Rahandi Lubis

**Affiliations:** 1 Department of Forest Products, Faculty of Forestry, Universitas Sumatera Utara, Medan 20155, Indonesia; 2 Forest Product Department, Faculty of Forestry and Environment, IPB University, Bogor 16680, Indonesia; 3 Research Center for Biomass and Bioproducts, National Research and Innovation Agency, Cibinong 16911, Indonesia; 4 Research Collaboration Center for Biomass and Biorefinery between BRIN and Universitas Padjadjaran, Jatinangor 45363, Indonesia

**Keywords:** maleic acid, particleboard, pressing temperature and time, sorghum bagasse, table tennis blade

## Abstract

This physical and mechanical properties of a table tennis blade made from sorghum bagasse particleboard (TTBSB-particleboard) bonded maleic acid adhesive was investigated under pressing temperature and time variations. The TTBSB-particleboard was produced via a two-stage process in this study. A pressing temperature of 170–200 °C was used to prepare the first stage for 10 min. Following this, the second stage of the TTBSB-particleboard was produced with a different pressing time of 5–20 min. The TTBSB-particleboard had a specified target density of 0.6 g/cm^3^ and a size of 30 cm × 30 cm × 0.6 cm, respectively. For references concerning the tested quality of TTBSB-particleboard, the JIS A 5908-2003 standard has been used. For comparison, the commercial blades of Yuguan Wooden 1011 and Donic Original Carbo Speed were tested under the same conditions. The quality of the TTBSB-particleboard was successfully enhanced by increasing the pressing temperature (170 to 200 °C) and time (5 to 20 min). As a result, the pressing condition of 200 °C and 20 min were effective in this study. The TTBSB-particleboard in this study has a greater weight than the commercial blades of Yuguan and Donic. However, the TTBSB-particleboard in this study had a ball rebound comparable to that of the Donic blade.

## 1. Introduction

The blade is an essential component in table tennis. The main ingredients for making the blade consist of wood (plywood) and synthetic fibers. The woods commonly used as blades are kiri (*Paulownia tomentosa*), balsa (*Ochroma pyramidale*), ayous (*Triplochiton scleroxylon*), hinoki (*Chamaecyparis obtuse*), koto (*Pterygota macrocarpa*), and limba (*Terminalia superba*) [1]. Arifin et al. [1] reported that wood as a raw material for the blade has drawbacks. Namely, it is expensive compared to natural fibers, more challenging to produce, and more difficult to dry. In addition, the availability of wood as a raw material is decreasing due to deforestation. The MoEF reports that the size of deforestation in Indonesia from 2018 to 2019 was 0.46 million ha [2]. Making wood-based composites using natural fibers has been developed as one of the solutions to deforestation.

Natural fibers, such as kenaf, hemp, and sisal, can be used as a substitute for wood to manufacture table tennis blades. Arifin et al. [1] reported natural fibers, such as kenaf, hemp, and sisal, as a substitute for wood in manufacturing wood-based composite products, such as table tennis blades. This research showed that kenaf fiber is the best fiber substitute for wood in table tennis blades based on energy absorption, its lighter weight, shear strength, Young’s modulus, and hardness. Amin et al. [3] also reported that kenaf fiber can replace wood as the primary material in manufacturing table tennis blades.

Sorghum is another natural fiber source that has the potential to be used as a raw material for the production of blades. The sorghum harvest period is relatively short, around 3–4 months [4]. The stalks of sorghum plants have an average height of 1–3.4 m [4]. In addition, according to Pabendon et al. [5], the average bagasse of sorghum stalks is 23.06 tons/ha, while the average bagasse of sorghum bagasse is 4.89 tons/ha. Sorghum bagasse can be used as raw material for particleboard [6,7,8,9,10,11,12,13]. Particleboard made from sorghum bagasse has good physical and mechanical properties. According to the findings of Kusumah et al. [11,14] studies, the optimum conditions for producing sorghum bagasse particleboard with citric acid adhesive were at a 20% adhesive and a pressing temperature of 200 °C for 10 min. Sorghum bagasse particleboard under these conditions had a modulus of elasticity, internal bonding, and thickness swelling that met the standard JIS A 5908:2003 type 18. In addition, these qualities were comparable to particleboard made using phenol–formaldehyde (PF) and polymeric 4,4-methylene diphenyl diisocyanate (pMDI).

Another organic acid that can be used as an adhesive is maleic acid. Maleic acid was obtained by removing water from malic acid [15]. Sejati et al. [16] reported that maleic acid has a lower melting point, so maleic acid is more reactive than malic acid and citric acid, resulting in higher dimensional stability, strength, and durability in wood modification. According to a prior study, sorghum bagasse particleboard bonded with maleic acid adhesive performed well and met the requirements of JIS A 5908-2003 type 8 [17]. These characteristics are comparable to the citric acid-bonded particleboard and offer greater resistance to termites and decay than phenol–formaldehyde adhesives.

Sutiawan et al. [18] investigated the maleic acid content and particle size on a table tennis blade made from sorghum bagasse particleboard (TTBSB-particleboard). The performance of TTBSB-particleboard improved with an increasing maleic acid content of 15 wt%. The powder particle class (4–20 mesh) provided higher dimensional stability, internal bonds, and smoother TTBSB-particleboard than the coarse particle class (4–10 mesh) due to the more significant contact area among the powder particle class. However, the mixed particle class (4–20 mesh) has an optimal modulus of elasticity and rupture of the TTBSB-particleboard. Therefore, the maleic acid content of 15 wt% and mixed particle class resulted in optimum physical and mechanical properties for the TTBSB-particleboard [18].

The parameters of pressing temperature and time play an essential role in determining the rate of curing of the adhesive [19]. Muruganandam et al. [20] stated that the temperature and time of the press depend on the type of raw material used and the product produced. In addition, the hot-pressing process is important in manufacturing boards because of the energy expenditures required and the impact of pressing time on productivity [20]. Therefore, in this study, the effect of pressing temperature and time on the quality of table tennis blade sorghum bagasse particleboard (TTBSB-particleboard) was examined and compared with the commercial table tennis blade. The quality of the TTBSB-particleboard was determined at effective pressing temperatures and times. The novel idea from this research is to manufacture a new table tennis blade made from sorghum bagasse as the main material to replace wood.

## 2. Material and Methods

### 2.1. Materials

Sorghum bagasse (*Sorghum bicolor*) from a garden at the National Research and Innovation Agency (BRIN) was utilized, referring to [18]. Pure maleic acid (MA) was bought from the Telagasakti Skautama Company (Jakarta, Indonesia). In addition, the commercial blade of a Donic Original Carbo Speed (Berlin, Germany) and Yuguan Wooden 1011 (Beijing, China) were bought from Asta Sport Company (Jakarta, Indonesia).

### 2.2. Sample Preparation

In accordance with earlier articles, a chipper and a knife-ring flaker machine were used to create the sorghum bagasse particles [18]. Sorghum bagasse particles were sieved according to optimum conditions per a previous study at 4–20 mesh (mixed particle size class) [18]. After that, the material was dried at 60 °C until the moisture content was at 5%. According to previous research, MA and water were set at 44 wt% in water [17].

### 2.3. Manufacture of TTBSB-Particleboards

A table tennis blade made from sorghum bagasse particleboard (TTBSB-particleboard) was produced via a two-stage method in this study. The first stage was prepared at different pressing times of 170–200 °C for 10 min. The influence of pressing temperature was evaluated to obtain the optimum pressing temperature for TTBSB-particleboard production. Then, another stage of TTBSB-particleboard was manufactured for a different pressing time at 5–20 min. Table 1 presents the stage production of the TTBSB-particleboard in this study. The TTBSB-particleboard had a specified target density of 0.6 g/cm^3^ and a size of 30 cm × 30 cm × 0.6 cm, respectively. Before pressing, the moisture content was decreased by a 12 h oven treatment at 80 °C [11]. The pressing pressure was set at 5 MPa for producing TTBSB-particleboard, according to [11].

### 2.4. Evaluation of the Properties of TTBSB-Particleboards

The JIS A 5908:2003 and D 1037:1999 standards were used for testing the physical and mechanical characteristics of the TTBSB-particleboard with some modifications [21,22]. The parameters of physical properties tested consisted of density, moisture content (MC), water absorption (WA), and thickness swelling (TS), and the mechanical properties tested consisted of modulus of elasticity (MOE), modulus of rupture (MOR), internal bonding (IB), surface roughness (SR), and hardness (H).

The samples used for the density test were 5 × 5 × 0.6 cm^3^ in terms of length, width, and thickness, respectively. Comparing the mass and volume of TTBSB-particleboards revealed the density determination. The MC test was conducted using a sample of the size 5 × 5 × 0.6 cm^3^. Following a 24 h drying period in an oven set to 105 °C, the MC test was determined using the initial and final masses. The WA and TS of the TTBSB-particleboard were determined using a 24 h immersion test with a 5 × 5 × 0.6 cm^3^ specimen. The MOE and MOR were determined using the universal testing machine (UTM AGX, Shimadzu, Japan) with dimensions, spans, and loading speeds of 15 × 3 × 0.6 cm^3^, 9 cm, and 10 mm/min, respectively. The same size for TS and WA was utilized for testing the IB of the TTBSB-particleboard.

Using a portable tester (SJ-210, Mitutoyo, Japan), the 50 × 50 × 6 mm^3^ specimens were examined to measure the surface roughness (average roughness/Ra) of the TTBSB-particleboard [23]. The specimens were put through a hardness test after evaluating the surface roughness. In addition, the hardness was assessed using the ball test at a speed of 6 mm/min with a projected area of 100 mm^2^. Hardness is measured by the stress at which the ball penetrates to a depth of half its diameter. Six replications were used for the TTBSB-particleboard experiment testing. Using the universal testing machine (UTM AGX, Shimadzu, Japan), the hardness was investigated according to the standard ASTM D143.

**Figure 1:**
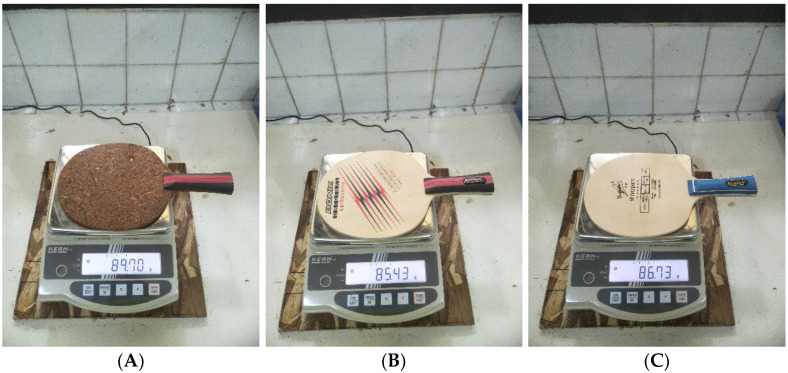
TTBSB-particleboard prepared at 200 °C for 20 min, TTBSB-Particleboard (**A**), Donic blade (**B**), and Yuguan blade (**C**).

In addition, weight and ball rebound were tested in this study. Weight and all TTBSB-particleboards were tested in previous studies [24]. Here, the TTBSB-particleboard weight was measured using a digital scale. Meanwhile, TTBSB-particleboard is too high to allow for measuring the height of the first ball to be applied at 90 cm. For comparison, the commercial blade of the Donic Original Carbo Speed (Germany) and Yuguan Wooden 1011 (China) was tested under the same conditions (Figure 1).

### 2.5. Fourier Transform Infrared Spectroscopy (FTIR) Analysis

A universal attenuated total reflectance (UATR) accessory on a Fourier transform infrared (FTIR) instrument (Spectrum Two, PerkinElmer, USA) was employed to determine changes in the functional groups, and it was used in absorbance mode. The FTIR spectra at 4 cm^−1^ were recorded in the spectral region 4000 to 400 cm^−1^. The spectra were corrected for baseline using PerkinElmer software (Ver. 10.5.3, Perkin Elmer Inc., Hopkinton, MA, USA) [19].

### 2.6. X-ray Diffraction (XRD) Analysis

The crystallinity of the sorghum bagasse and TTBSB-particleboard were determined using X-ray diffraction. The PerkinElmer XD-2 (4000, PerkinElmer, USA) was used to acquire the X-ray diffraction data for this study. At a scan rate of 2°/min, data on X-ray scattering were collected over a 10° to 60° 2θ range [25].

### 2.7. Statistical Analysis

A simple, utterly randomized design with one factor from each stage was evaluated using an analysis of variance (ANOVA). Then, Duncan’s multiple range test was performed to statistically analyze the difference in TTBSB-particleboard properties at α < 0.05.

## 3. Result and Discussion

### 3.1. Pressing Temperature

The water absorption (WA) and thickness swelling (TS) of TTBSB-particleboard manufactured at different pressing temperatures are presented in Table 2. In this investigation, a considerable drop in temperature of up to 200 °C was discovered on the WA and TS of TTBSB-particleboard. The TS of TTBSB-particleboard met the requirement (12%), thus, following the JIS A 5908 standard. The result showed that the TTBSB-particleboard manufactured in this study had good dimensional stability. Kusumah et al. [11] reported that the cross-linking between organic acid-based adhesives and sorghum bagasse particles at temperatures above 200 °C might form faster than at temperatures below 180 °C. Therefore, the hypothesized results showed improved cross-linking in the TTBSB-particleboard.

Table 2 shows the MOE and MOR of the TTBSB-particleboard at different pressing temperatures. With the increase in pressing temperature from 170 to 200 °C, the MOE and MOR of the TTBSB-particleboard were increased. At 200 °C pressing temperature, the maximum average MOE and MOR values of TTBSB-particleboard were 440 MPa and 4.75 MPa, respectively. These values were approximately 2.5 times or more significant than those obtained with TTBSB-particleboard at 170 °C. In terms of MOE and MOR in this study, the effective pressing temperature was 200 °C. As with those previously reported by Kusumah et al. [14], these phenomena demonstrated that the effective pressing temperature of particleboard bonded with organic acid-based adhesives, such as citric acid, is 200 °C.

The increasing temperature from 170–200 °C increased the IB of the TTBSB-particleboard (Table 2). The TTBSB-particleboard manufactured at 200 °C had a threefold higher IB than TTBSB-particleboard manufactured at 170 °C. Furthermore, temperature and adhesive curing were observed to have a linear correlation [26]. According to the general theory, the melting point of MA is around 130 °C. Therefore, the excellent TTBSB-particleboard manufactured at 200 °C was found due to the MA melting and could have easily created linkages between sorghum bagasse particles. Additionally, the IB TTBSB-particleboard met the JIS A 5908 standard (0.15 MPa) requirements at 190 °C and 200 °C in this study.

### 3.2. Pressing Time

The TTBSB-particleboard produced under the pressing temperature of 200 °C in the previous study was the optimum TTBSB-particleboard. This was due to good physical and mechanical properties and, thus, at this stage, we analyzed the effect of pressing time and compared the TTBSB-particleboard with commercial blades, such as the Yuguan Wooden 1011 (China) and Donic Original Carbo Speed (Germany) (Table 3).

Density is a physical property that affects the quality of table tennis blades [27]. The average density of TTBSB-particleboard in this study is 0.55–0.57 g/cm^3^, and is lower than the Yuguan commercial blade of 0.67 g/cm^3^, but higher density than the Donic commercial blade (0.42 g/cm^3^). Differences in density are due to the composition of the materials used. The core part of the TTBSB-particleboard is made of particleboard, while the Donic commercial blade is made of solid wood and a combination of veneer. Manin et al. [24] stated that in the arrangement of table tennis blades, low-density wood is used as the blade’s core, while high-density wood is used as the blade’s surface. In addition, the average moisture content (MC) of TTBSB-particleboard in this study (8.93–11.31%) was lower than that of the Yuguan commercial blade (10.74%) but higher than that of the Donic commercial blade (6.53%).

Another physical property affecting table tennis blades’ quality is dimensional stability [27]. Dimensional stability can be evaluated by testing water absorption (WA) and thickness swelling (TS) [21]. The lowest WA and TS values for TTBSB-particleboard in this study were found at a pressing time of 20 min. The TTBSB-particleboard had a TS value of 3.01% after 20 min of pressing. These values differed significantly (*p* < 0.05) from those obtained during another pressing time. The TTBSB-particleboard has a higher WA than the Yuguan commercial blade and Donic commercial blade. This is because the density of particleboard in this study is medium (0.6 g/cm^3^). Liao et al. [25] reported that the WA decreased with increasing density, indicating that water penetration into the board was prevented by higher density. However, the TS of the TTBSB-particleboard in this study (3.01–4.26%) was comparable compared to the Yuguan commercial blade (4.25%) and the Donic commercial blade (4.93%).

Modulus of elasticity (MOE) and modulus of rupture (MOR) were the mechanical properties that affected the quality of table tennis blades. The greater the modulus of elasticity and rupture of a table tennis blade, the stronger the blade is in response to deformation and for producing an accurate ball rebound [28]. The pressing time from 5 to 20 min resulted in increasing MOE of the TTBSB-particleboard (*p* < 0.05). Meanwhile, for the MOR of TTBSB-particleboard, the value gradually increased from 5 to 15 min and then decreased slightly at 20 min (*p* >0.05). Therefore, in terms of MOE and MOR in this study, an effective pressing time of 20 min was determined. The MOE and MOR of TTBSB-particleboard in this study were lower than for Yuguan commercial blades and Donic commercial blades (Table 3). The high MOR of Donic’s commercial blades is due to carbon fiber with a high density of 1.9 g/cm^3^ [29]. Sun et al. [29] and Wang et al. [27] reported that using carbon fiber increased the strength of table tennis blades.

Manin et al. [24] stated that bonding quality is an important component affecting table tennis blades’ quality. Bonding quality in TTBSB-particleboard can be evaluated using internal bonding (IB) [21]. The increasing pressing time has resulted in increased IB of TTBSB-particleboard manufactured at 5–20 min (*p* < 0.05). The 15 min (0.21 MPa) and 20 min (0.22 MPa) pressing time had a significantly higher IB (*p* < 0.05), 1.5 times that of the TTBSB-particleboard after 5 min (0.15 MPa) and 10 min (0.15 MPa). However, the IB of TTBSB-particleboard after 15 min and 20 min pressing time was similar (*p* > 0.05). In this study, the IB of Yuguan and Donic commercial blades was higher than for the TTBSB-particleboard. This is possibly because commercial blades utilized isocyanate adhesives and carbon fiber in their components. Sun et al. [29] and Wang et al. [27] reported that using carbon fiber increased the strength of table tennis blades.

Surface roughness and hardness affect the quality of table tennis blades. The surface roughness and hardness affect the rebound of the table tennis ball [27]. The TTBSB-particleboard used in this investigation has a 4.86 to 5.48 µm surface roughness and is higher than the Yuguan commercial blade (2.10 µm) and Donic commercial blade (0.92 µm). The high surface roughness is because the TTBSB-particleboard has not been sanded. In addition, the hardness of the TTBSB-particleboard in this study was 29.77–32.30 MPa and was comparable to the Yuguan commercial blade (38.69 MPa) and the Donic commercial blade (25.89 MPa).

The TTBSB-particleboard in this study has a greater weight than the commercial blades of Yuguan and Donic. Iino and Kojima [30] reported that the weight of the blade does not affect the performance of a table tennis player, but the weight of the blade determines the type of play of the player. The dominant type of player generally uses heavy blades to defend. The TTBSB-particleboard in this study had a ball rebound comparable to that of Donic’s blade, which already had carbon (Table 3). The use of carbon in table tennis blades can improve the quality of the blade as well as its ball rebound. Sun et al. [29] reported that carbon has a relatively high price. The use of TTBSB-particleboard certainly has advantages because, in addition to utilizing a by-product in the form of sorghum bagasse, which is not used optimally, it also has a good ball rebound.

### 3.3. FTIR Analysis

**Figure 2:**
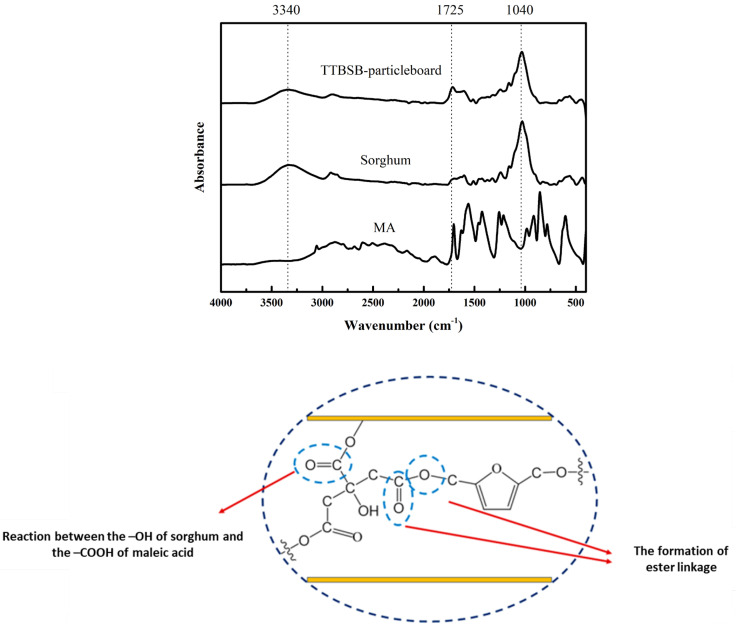
FTIR spectra of maleic acid adhesive, sorghum bagasse, and TTBSB-particleboard prepared at 200 °C for 20 min, and their scheme reaction.

Figure 2 illustrates the FTIR spectrum of sorghum and TTBSB-particleboard prepared at 200 °C for 20 min and their scheme reaction. The TTBSB-particleboard has a lower hydroxyl group (-OH) concentration than sorghum at 3340 cm^−1^ [31]. These findings imply that the MA and the -OH group on cellulose molecules interacted to produce ester bonds. According to these findings, the dimensional stability of the TTBSB-particleboard was higher. Additionally, the peak of TTBSB-particleboard samples was greater than that of sorghum at approximately 1725 cm^−1^. According to Sutiawan et al. [17], the reaction between the carboxyl group of MA and the hydroxyl group of sorghum produces a specific spectral peak at 1700 cm^−1^, which is attributable to a double bond ester.

### 3.4. XRD Analysis

**Figure 3:**
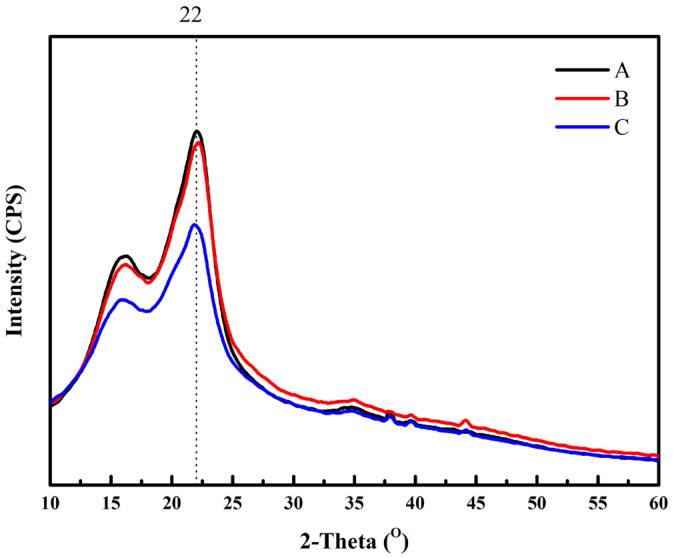
XRD spectra of (**A**) sorghum bagasse, (**B**) TTBSB-particleboard prepared at 180 °C for 10 min, and (**C**) TTBSB-particleboard prepared at 200 °C for 20 min.

The X-ray diffraction spectra of sorghum and TTBSB-particleboard are shown in Figure 3. The crystallinity of sorghum bagasse (26.22%) was higher than TTBSB-particleboard at 180 °C for 10 min (24.27%) and TTBSB-particleboard at 200 °C for 20 min (23.48%). The use of MA for TTBSB-particleboard reduces crystallinity. Fatriasari et al. [32] reported that MA pre-treatment turns more crystalline fractions, such as cellulose, into amorphous fractions and lowers the degree of crystallinity. This might be another reason why the MOR TTBSB-particleboard at 20 min was slightly lower than at 15 min.

## 4. Conclusions

The quality of the TTBSB-particleboard was affected by pressing temperature and time. The quality of the TTBSB-particleboard was successfully enhanced by increasing the pressing temperature from 170 to 200 °C for 10 min. Additionally, when the pressing time was increased from 5 to 20 min, the quality of the TTBSB-particleboard increased. As a result, the pressing condition of 200 °C and 20 min were effective in this study. The thickness swelling and hardness of the TTBSB-particleboard in this study were comparable to the Yuguan commercial blade and the Donic commercial blade. The TTBSB-particleboard in this study had a greater weight than the commercial blades of Yuguan and Donic. However, the TTBSB-particleboard in this study had a ball rebound comparable to that of Donic’s blade, which already contained carbon. The use of TTBSB-particleboard certainly has advantages because, in addition to utilizing a by-product in the form of sorghum bagasse, which is not utilized optimally, it also has a good ball rebound.

## Figures and Tables

**Table 1:** Conditions of manufacture for TTBSB-particleboard.

Stages	Pressing Temperature (°C)	Pressing Time (min)
Stage 1	170	10
	180	10
	190	10
	200	10
Stage 2	200	5
	200	10
	200	15
	200	20

**Table 2:** Properties of TTBSB-particleboards at different pressing temperatures.

Properties	Pressing Temperature (°C)			
	170	180	190	200
Water Absorption (WA) (%)	89.67 (7.28) b	81.67 (7.76) b	85.19 (3.48) b	72.03 (6.63) a
Thickness Swelling (TS) (%)	6.13 (2.02) b	4.61 (0.69) a	4.85 (0.61) ab	3.89 (0.58) a
Modulus of Elasticity (MOE) (MPa)	175 (84.33) a	237 (163.97) a	372 (86.78) b	440(64.41) b
Modulus of Rupture (MPa)	1.98 (0.52) a	2.41 (0.42) a	4.31 (0.58) b	4.75 (0.70) b
Internal Bonding (IB) (MPa)	0.03 (0.02) a	0.11 (0.04) b	0.15 (0.04) c	0.16 (0.05) c

Values in parentheses are standard deviations. According to Duncan’s multiple range test, values are followed by the symbols a and b if the same letter is not statistically different (*p* > 0.05).

**Table 3:** Properties of TTBSB-particleboards at different pressing times.

Properties	Pressing Time (min)				Commercial Blade	
	5	10	15	20	Donic	Yuguan
Density (g/cm^3^)	0.57 (0.02) a	0.57 (0.05) a	0.56 (0.01) a	0.55 (0.01) a	0.42 (0.00)	0.67 (0.22)
Moisture Content (MC) (%)	11.31 (0.18) b	9.90 (1.73) a	9.19 (0.21) a	8.93 (0.10) a	6.53 (0.08)	10.74 (0.27)
Water Absorption (WA) (%)	92.19 (5.28) b	94.75 (5.83) b	89.54 (9.02) ab	81.76 (5.96) a	39.33 (1.84)	68.75 (5.37)
Thickness Swelling (TS) (%)	4.26 (1.52) a	3.75 (0.57) a	3.89 (1.32) a	3.01 (0.25) a	4.93 (0.40)	4.25 (0.83)
Modulus of Elasticity (MOE) (MPa)	611 (163) a	758 (115) ab	783 (210) ab	822 (118) b	2752 (583)	4485 (454)
Modulus of Rupture (MPa)	3.20 (0.88) a	4.90 (0.55) b	4.67 (1.22) b	4.54 (0.63) b	63.79 (2.85)	49.04 (5.60)
Internal Bonding (IB) (MPa)	0.15 (0.04) a	0.15 (0.04) a	0.21 (0.05) b	0.22 (0.04) b	0.98 (0.04)	2.36 (0.09)
Surface Roughness (SR) (µm)	4.86 (0.90) a	5.00 (1.26) a	5.48 (0.92) a	5.39 (0.81) a	0.92 (0.03)	2.10 (0.53)
Hardness (H) (MPa)	29.77 (2.90) a	31.76 (2.37) a	32.30 (1.59) a	31.49 (1.36) a	25.89 (0.28)	38.69 (3.07)
Weight (g)	-	-	-	89.70	85.43	86.73
Ball Rebound (cm)	-	-	-	54.67 (1.52)	58.67 (0.58)	55.67 (1.53)

Values in parentheses are standard deviations. According to Duncan’s multiple range test, values followed are by symbols a and b if the same letter is not statistically different (*p* > 0.05).

## Data Availability

The data presented in this study are available on request from the corresponding author.
